# Artificial intelligence vs. human expert: Licensed mental health clinicians' blinded evaluation of AI-generated and expert psychological advice on quality, empathy, and perceived authorship

**DOI:** 10.1016/j.invent.2025.100841

**Published:** 2025-06-03

**Authors:** Ludwig Franke Föyen, Emma Zapel, Mats Lekander, Erik Hedman-Lagerlöf, Elin Lindsäter

**Affiliations:** a Division of Psychology, Department of Clinical Neuroscience, Karolinska Institutet, Stockholm, Sweden; b Stress Research Institute, Department of Psychology, Stockholm University, Stockholm, Sweden; c Gustavsberg University Primary Care Center, Stockholm Health Care Services, Region Stockholm, Sweden; d Osher Center for Integrative Health, Department of Clinical Neuroscience, Karolinska Institutet, Stockholm, Sweden; e Division of Clinical Psychology, Department of Psychology, Uppsala University, Uppsala, Sweden; f Center for Psychiatry Research, Department of Clinical Neuroscience, Karolinska Institutet, Stockholm Health Care Services, Region Stockholm, Sweden

**Keywords:** Artificial intelligence, Digital health, Empathy, Mental health, Psychological advice, Therapeutic Alliance

## Abstract

**Background:**

The use of artificial intelligence for psychological advice shows promise for enhancing accessibility and reducing costs, but it remains unclear whether AI-generated advice can match the quality and empathy of experts.

**Method:**

In a blinded, comparative cross-sectional design, licensed psychologists and psychotherapists assessed the quality, empathy, and authorship of psychological advice, which was either AI-generated or authored by experts.

**Results:**

AI-generated responses were rated significantly more favorable for emotional (OR = 1.79, 95 % CI [1.1, 2.93], *p* = .02) and motivational empathy (OR = 1.84, 95 % CI [1.12, 3.04], *p* = .02). Ratings for scientific quality (*p* = .10) and cognitive empathy (*p* = .08) were comparable to expert advice. Participants could not distinguish between AI- and expert-authored advice (*p* = .27), but *perceived* expert authorship was associated with more favorable ratings across these measures (ORs for perceived AI vs. perceived expert ranging from 0.03 to 0.15, all *p* < .001). For overall preference, AI-authored advice was favored when assessed blindly based on its actual source (*β* = 6.96, *p* = .002). Nevertheless, advice *perceived* as expert-authored was also strongly preferred (*β* = 6.26, *p* = .001), with 93.55 % of participants preferring the advice they believed came from an expert, irrespective of its true origin.

**Conclusions:**

AI demonstrates potential to match expert performance in asynchronous written psychological advice, but biases favoring perceived expert authorship may hinder its broader acceptance. Mitigating these biases and evaluating AI's trustworthiness and empathy are important next steps for safe and effective integration of AI in clinical practice.

## Highlights

• Clinicians rated AI-advice as equally or more empathetic and sound than expert-written advice.
• Clinicians could not reliably distinguish between AI- and expert-authored psychological advice.
• Perceived authorship influenced ratings, with expert-attributed responses receiving higher scores.
• Findings highlight potential biases in the acceptance of AI-generated mental health support.

## 1 Background

The integration of artificial intelligence (AI) for psychological advice has evolved significantly since the creation of ELIZA (Weizenbaum, 1966), a conversational agent designed to simulate Rogerian psychotherapy by mimicking reflective, non-directive responses. ELIZA was a far cry from the sophisticated systems available today, but it already demonstrated the potential of AI to engage users and sparked interest in conversational agents. The so-called “ELIZA effect,” where users attributed human-like qualities to the chatbot (Tarnoff, 2023), highlighted the compelling nature of such interactions. Today's large language models (LLMs) and natural language processing (NLP)-based conversational agents were not originally designed to provide psychological advice, but researchers are investigating their potential to do so. With up to 70 % of individuals with mental illness lacking adequate care (Boucher et al., 2021), modern AI tools have been proposed to address resource shortages by providing timely, personalized support (Egan et al., 2024; Moitra et al., 2023). Preliminary evidence on the effectiveness of conversational agents in mental health is mixed. A meta-analysis of 12 studies found weak evidence for improvements in symptoms of depression, stress, and specific phobias after using AI-based treatments, while underscoring significant risk of bias and the lack of high-quality trials (Abd-Alrazaq et al., 2020). Individual interventions, such as “Tess,” using natural language processing (NLP) and emotion detection, significantly reduced depressive and anxiety symptoms and outperformed bibliotherapy (Fulmer et al., 2018). Similarly, conversational agents like “Woebot” and “Wysa” have demonstrated potential in alleviating symptoms of depression and anxiety, particularly among highly engaged users (Fitzpatrick et al., 2017; Inkster et al., 2018).

While conversational agents have demonstrated potential to engage users while delivering support (Boucher et al., 2021), significant questions remain about their suitability in acting as substitutes for therapists. Limitations in scientific depth and accuracy of AI-generated responses persist, with some studies finding notable gaps in their factual reliability (Ma et al., 2023). However, another study showed that, when blinded, AI-generated responses to questions about mental health problems were rated more authentic, professional, and practical compared to expert-authored responses (Lopes et al., 2023). It is noteworthy that while linguistic patterns in AI responses often lack diversity, even linguistic experts struggle to consistently identify AI-generated text, achieving only 38.9 % accuracy (Desaire et al., 2023).

Research has shown that trust and acceptance of AI in healthcare contexts are strongly influenced by human-like attributes, perceived empathy and interaction quality (Pelau et al., 2021; Stade et al., 2024). Cognitive empathy, or understanding and acknowledging others feelings, has been suggested to be particularly impactful, as it fosters therapeutic alliance and helps practitioners better understand and respond to patients' emotions (Moudatsou et al., 2020). While there has been some skepticism around empathy in AI, some studies have suggested that AI-generated responses can be perceived as highly empathetic. Ayers et al. (2023), for example, found that users rated AI-generated mental health responses as more empathetic than those written by experts. However, other research indicates that users still prefer expert responses for their perceived authenticity and depth (Morris et al., 2018). It is worth noting that such findings may reflect the limitations of earlier-generation models. Given the rapid pace of development in conversational agents, results from studies conducted just a few years ago—such as Morris et al. (2018)—may no longer be representative of current capabilities. As the technology continues to evolve, specifying the exact model and version under evaluation becomes increasingly important. Emerging research indicates that newer models are better able to capture linguistic nuance and contextual cues, which may contribute to enhanced perceptions of empathy (Li et al., 2024). In sum, while conversational agents have demonstrated potential to engage users and deliver support, significant questions remain about its performance and suitability in acting as a substitute for a therapist. There remain critical concerns about ethical implications of deploying conversational agents in sensitive healthcare contexts, where errors or misunderstandings could harm users (Coghlan et al., 2023). As researchers continue to refine these systems, it will be crucial to examine if AI-generated advice can be of similar quality as expert advice in terms of scientific quality and empathy, and to investigate whether the perceived source of the advice affects its perceived quality.

### 1.1 Objective of the study

This explorative study investigates the quality and empathy of AI (based on a LLM, GPT-4) generated written psychological advice as compared to expert advice using ratings by licensed mental health clinicians. It also examines their ability to distinguish between AI and expert-authored advice, and how perceived authorship influences ratings of quality and empathy. By focusing on clinicians' evaluations, this study aims to provide a qualified assessment of AI-generated content and identify potential biases that may affect trust in AI for psychological advice.

### 1.2 Research questions

I. Can AI produce psychological advice that is comparable to advice by an expert in terms of scientific quality and empathy?
II. To what extent are clinicians able to identify whether psychological advice was produced by an AI or an expert?
III. Does the clinicians' perception of authorship relate to their preference and scores of the level of empathy, and scientific quality of the advice?

## 2 Methods

### 2.1 Study design

The study employed a cross-sectional design comparing psychological advice generated by a conversational agent to that given by psychologists, psychotherapists and psychiatrists published in an advice column in a Swedish national newspaper. Published reader questions around mental health and advice by experts were the basis for the study. Participants were presented with randomly selected question and response sets, blinded to authorship and subsequently asked to rate them. The study was pre-registered on Open Science Framework (Zapel et al., 2024).

### 2.2 Participants

Participants were recruited using a convenience sampling method, targeting Swedish-speaking licensed mental health clinicians, specifically licensed psychologists, psychotherapists and physicians (hereafter referred to as clinicians). Recruitment was conducted via multiple channels, including clinic posters, targeted email outreach and social media forums aiming to maximize sample diversity.

A total of 47 participants were recruited, of which four were excluded (two non-completers, two not meeting criteria), resulting in a final sample of 43 licensed mental health clinicians (40 psychologists and three psychotherapists). No sensitive data were collected and participation in the study was anonymous.

### 2.3 Materials

#### 2.3.1 Newspaper articles

The study used 26 mental health advice columns from ‘Dagens Nyheter,’ a major Swedish newspaper (https://www.dn.se/) published between January 2020 and January 2024. These columns consisted of reader questions on issues like relationship problems and mental health problems and similar topics and the corresponding responses from psychologists, psychotherapists or psychiatrists employed by the newspaper to provide written advice. Columns were chosen by (EZ) to represent a variety of topics and only from the most recent newspaper issues to limit the possibility of AI familiarity with the articles. Of the chosen advice columns, 77 % (*n* = 20) were used to train a chatbot, while 23 % (*n* = 6) were included in a survey. These articles were fairly comprehensive with a median length of the reader questions of 340 words, and the expert responses had a median length of 834 words and were written by four different authors with a coincidental predominance of one specific author (53 % of all, 67 % of the test set).

#### 2.3.2 The conversational agent

The conversational agent was developed using OpenAI's model GPT-4 (Fråga Insidan, 2024). It was enhanced using retrieval augmented generation and a training set of 20 of the mental health advice columns. The conversational agent had at the time of article generation been trained on data up until early 2023 and could not have been exposed to five out of six articles in the test set. The conversational agent was instructed to mimic the style and tone of a professional psychologist writing for a Swedish advice column. For the specific prompt instructions, see Zapel et al. (2024). For the purpose of this study, the conversational agent was not directly used by participants but the answers were extracted and slightly adjusted with minor edits by the research team to remove specific signs of AI writing (like bullet points), to allow for blinded comparison with expert answers.

#### 2.3.3 The rating questionnaire

The questionnaire was created on ‘Google Forms’. It included the six reader questions and expert answers from the newspaper combined with the alternative answer generated by the conversational agent. Out of six sets, each participant was presented with two randomly selected sets. Participants rated the texts for empathy and scientific quality (scale of 1–5), perceived authorship (AI or Expert), and preference (binary). After having completed two sets, participants were offered to rate the additional four sets. In total, the questionnaire contained seven sections and 73 items; each participant answered a minimum of 26 items, with three questions assessing inclusion criteria and 11 questions per article set. The questions were constructed by the researchers and included a description for each construct investigated (for the complete questionnaire see: Zapel et al., 2024).

#### 2.3.4 Outcome measurements and variables

Subjective ratings from participants, all licensed psychologists and or psychotherapists, served as outcome measures. No personal demographics were collected beyond professional background and Swedish language proficiency.

The study's independent variable was authorship (AI-generated vs. expert-written), while dependent variables included scientific quality and empathy (ordinal scales; 1 = very poor, 5 = very high) as well as perceived authorship and preference (binary).

Scientific quality was rated using a single item assessing the overall scientific soundness of the advice.

Empathy was assessed using 3 items based on the components suggested by Montemayor et al. (2022): emotional empathy, cognitive empathy, and motivational empathy. Emotional empathy was defined as “the ability to share and understand the feelings of others as if they were one's own. Cognitive empathy was defined as “the capacity to understand and acknowledge the perspectives and feelings of others without necessarily sharing them.” Motivational empathy was defined as “the drive to respond appropriately to someone's emotional state or needs, often leading to supportive or helping behaviors.” This 3-item empathy scale demonstrated good internal consistency in the current study (based on *N* = 208), with a Cronbach's alpha of 0.89 and an average inter-item correlation of 0.74.

### 2.4 Statistical analysis

The statistical analysis plan for this study was pre-registered (Zapel et al., 2024), and an initial power analysis determined that a sample of 64 respondents was required to detect a medium effect size (*d* = 0.5) with 80 % power at the 0.05 alpha level for ordinal ratings, and over 95 respondents for binary outcomes. However, data collection was terminated earlier than planned due to monetary and time constraints. As a result, post-hoc adjustments were made to the analysis plan to improve robustness, including accounting for the nested structure of the data.

The primary analysis focused on the ordinal ratings of scientific quality and empathy, which were analyzed using cumulative link mixed models. Binary outcomes, such as preference (AI vs. expert), were modeled using generalized linear mixed models with a logit link function. Both types of models included random intercepts for raters and articles to account for variability at the individual and article level. The main fixed effects were actual authorship (AI vs. expert) and perceived authorship (participant's guess of authorship), with an interaction term included to investigate how these two factors influenced preferences and ratings. All statistical tests were two-sided and the alpha level was set at 0.05.

The analysis plan was adapted post-registration to include random effects for both raters and articles, acknowledging the hierarchical nature of the data where each rater assessed multiple articles and each article received multiple evaluations. Likelihood ratio tests were employed for model comparison and to assess the significance of interaction effects and the inclusion of random effects. Simplified models were also tested to isolate the main effects of perceived authorship and actual authorship on preferences.

Data extraction and cleaning were performed using Python (Van Rossum and Drake, 2010), utilizing libraries such as NumPy (Harris et al., 2020) and SciPy (Virtanen et al., 2020), with statistical analyses carried out in R (RStudio Team, 2024). The statistical code, along with the rating questionnaire and AI chatbot transcripts, is available on the Open Science Framework (OSF) for reproducibility and transparency (Zapel et al., 2024).

## 3 Results

Forty-three licensed mental health clinicians rated a total of 208 responses and 104 pairs. On average, each participant rated 2.4 response pairs (range: 1–6). As shown in Table 1, AI-generated advice received equal or more favorable ratings across all measures. For scientific quality (*p* = .10) and cognitive empathy (*p* = .08), the differences were not statistically significant, but AI responses were rated significantly more favorable for emotional empathy (*β* = 0.59, *p* = .02) and motivational empathy (*β* = 0.61, *p* = .02). Given that we did not reach the intended sample size, we conducted a post-hoc minimal detectable effect size analysis for the non-significant results. The analysis indicated that the study could detect effects of moderate size (OR ≥ 1.6–1.7), but lacked sensitivity to smaller effects which may explain the absence of statistically significant findings (Code available at Zapel et al. (2024).

**Table 1:** Ratings of AI and expert psychological advice to reader questions in a newspaper advice column.

		*M (SD)*				
Measure	Range	AI	Expert	*z-value*	Effect size (*OR* with 95 % *CI*)	*p-*value
Scientific Quality	(1–5)	3.46 (0.93)	3.21 (1.12)	1.65	1.53 (0.92, 2.53)	0.10
Emotional Empathy	(1–5)	3.45 (1.16)	3.05 (1.28)	2.33	1.79 (1.1, 2.93)	**0.02**
Cognitive Empathy	(1–5)	3.67 (1.01)	3.39 (1.14)	1.73	1.55 (0.94, 2.56)	0.08
Motivational Empathy	(1–5)	3.66 (1.06)	3.27 (1.21)	2.40	1.84 (1.12, 3.04)	**0.02**

*Note*. Raters were blinded to the true authorship of the advice.

Clinicians were unable to reliably distinguish between AI- and expert-authored responses, performing at chance level (45 % accuracy; *χ*^*2*^ test, *p* = .27). Answers *perceived* to be authored by an expert were rated more favorable across all measures of scientific quality and empathy, as shown in Table 2. Specifically, expert responses were rated more favorable in scientific quality (*β* = −1.89, *p* < .001), emotional empathy (*β* = −3.62, *p* < .001), cognitive empathy (*β* = −3.02, *p* < .001) and motivational empathy (*β* = −2.74, *p* < .001).

**Table 2:** Ratings of perceived AI and perceived expert psychological advice to reader questions in a newspaper advice column.

		*M (SD)*				
Measure	Range	Perceived AI	Perceived Expert	*z-value*	Effect size (*OR* with 95 % *CI*)	*p-*value
Scientific Quality	(1–5)	2.93 (0.99)	3.75 (0.91)	−6.28	0.15 (0.09, 0.24)	**< 0.001**
Emotional Empathy	(1–5)	2.42 (0.98)	4.12 (0.79)	−9.31	0.03 (0.01, 0.06)	**< 0.001**
Cognitive Empathy	(1–5)	2.91 (1.03)	4.19 (0.67)	−8.37	0.05 (0.03, 0.08)	**< 0.001**
Motivational Empathy	(1–5)	2.83 (1.14)	4.13 (0.70)	−8.06	0.06 (0.03, 0.11)	**< 0.001**

*Note*. Raters were blinded to the true authorship of the advice.

**Fig. 1:**
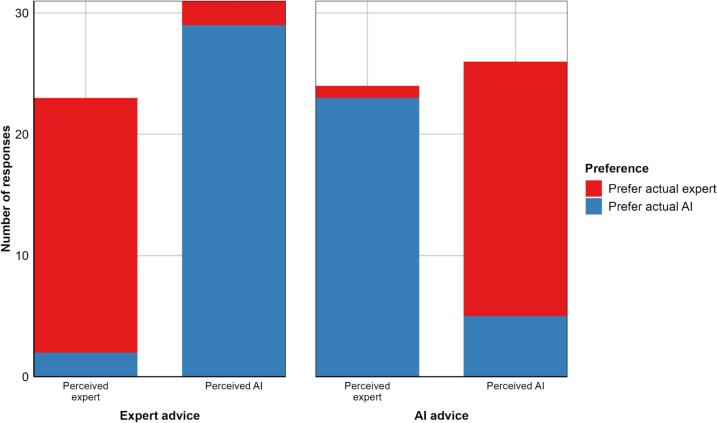
Preferences for AI and expert-authored advice by actual and perceived authorship *Note.* Preferences are displayed as the number of responses indicating a preference for AI or expert advice. Bars are grouped by actual authorship (Expert advice or AI advice) and further divided by perceived authorship (Perceived expert or Perceived AI).

The analysis of participants' preferences for AI- or expert advice revealed significant main effects for both actual authorship (*β* = 6.96, *p* = .002) and perceived authorship (*β* = 6.26, *p* = .001), indicating that AI-authored responses were preferred overall and that perceived expert authorship strongly influenced preferences. When participants perceived an answer as being authored by an expert, they overwhelmingly preferred that response, regardless of whether the answer was actually authored by AI or an expert (93.55 % preference for perceived expert advice). The interaction term between perceived and actual authorship was also significant (*β* = −12.29, *p* = .001), as visualized in Fig. 1 below.

## 4 Discussion

This cross-sectional study compared psychological advice provided by a conversational agent, developed using a LLM, GPT-4, to that given by experts in the context of a Swedish national newspaper advice column. The results of this study indicate that AI can produce psychological advice in a specific, structured, textual format comparable or even superior to psychological advice provided by experts with a high degree of scientific quality and empathy. Although the clinically trained raters were not able to distinguish between AI and expert advice they did display a preference for responses they perceived to be expert-authored when the original source was blinded. They also gave more favorable ratings of scientific quality and empathy when they thought the content was expert-authored. These results suggest a bias toward expert advice among clinically trained professionals, which could influence the acceptance of AI tools, despite AI's demonstrated ability to provide empathetic and high-quality support.

### 4.1 Quality

While findings for scientific quality in AI-writing have been mixed so far (Lopes et al., 2023; Ma et al., 2023) the present results suggest that AI may be suitable to provide psychological advice as the conversational agent tested in this study produced comparable or better results to experts. Notably, the training and outcome languages were Swedish, highlighting the potential for conversational agents to perform across diverse languages. These promising findings in a relatively small language suggest similar or better outcomes in languages with greater availability of training data, warranting further investigation.

### 4.2 Empathy

Empathy, as a critical component of effective psychological support, has traditionally been viewed as a uniquely human trait. Our results suggest that AI can offer written advice perceived to be highly empathetic, particularly displaying high levels of emotional and motivational empathy. This is surprising considering recent studies suggested that AI can only effectively mimic certain aspects of empathetic communication (Ayers et al., 2023). In this study, the prompts were relatively informative and slightly longer than average chatbot messages, which may have influenced the perceived empathy in AI responses as compared to other conversational agent formats.

### 4.3 Authorship - accuracy and influence of perceived authorship ratings

The accuracy of clinicians' guesses regarding the true source of the responses was approximately at chance level. This aligns with previous research, showing that professionals are generally unable to distinguish between support provided by experts and that generated by AI (Desaire et al., 2023). This inability, combined with the comparable scientific quality and empathy ratings between expert and AI-generated content, suggests a notable resemblance between the two sources of advice. However, when considering perceived authorship, the results show a clear bias: participants consistently rated content they believed was expert-authored more favorably. This supports previous findings (e.g., Jain et al., 2024), which suggest that perceived human expertise strongly impacts the selection and evaluation of psychological support, with clinicians tending to assume that experts offer superior advice. In particular, trust in the source appears to play a key role in how advice is received and acted upon (Shevtsova et al., 2024). While undisclosed AI advice is often seen as competent (Alowais et al., 2023) and can foster trust (Ayers et al., 2023), studies show that disclosing AI origins reduces perceived credibility and empathy, possibly leading to a nocebo effect in future AI interventions (Reis et al., 2024). However, other findings suggest that when patients knowingly interact with conversational agents, they may actually become more open and self-disclose more information, possibly due to reduced fear of judgment (Miner et al., 2017). User attitudes toward AI are shaped by factors like preexisting beliefs about AI competence and individual traits such as neuroticism (Vodrahalli et al., 2022; Bergdahl et al., 2023) but the strong effect of perceived authorship may also reflect well-established cognitive biases, such as authority bias—where humans tend to assign greater credibility to perceived experts—and algorithm aversion, a tendency to distrust machine-generated output after witnessing errors (Dietvorst et al., 2015). In this context, human-generated responses may be overvalued due to assumptions about empathy, intentionality, or experiential knowledge, while AI responses may trigger skepticism based in broader societal narratives about automation and technological control.

In sum, attitudes toward AI in mental healthcare warrant further investigation, especially as familiarity with AI grows, potentially influencing its acceptance and integration.

### 4.4 Implications for clinical practice

While the current results provide some evidence for AI's ability to provide written advice with high scientific quality and empathy, the absence of a genuine human relationship and the nuances of human interaction may limit the effectiveness of AI in a therapeutic context. A recent survey of community members and mental health professionals reflects the ambivalence surrounding AI's role in mental healthcare (Cross et al., 2024). While acknowledging its potential to improve accessibility, personalization, and efficiency, they voice concerns about compromised trust, risks to accuracy and ethics, and the loss of human connection central to the therapeutic relationship. These concerns are echoed by Carlbring et al. (2023), who emphasize that while AI may successfully simulate empathy, the establishment of therapeutic alliance and trust remains a significant challenge. Nevertheless, recent findings from Habicht et al. (2025) indicate that integrating a generative AI tool into group-CBT settings improved both patient engagement and clinical outcomes, suggesting the promise of conversational agents in such contexts. Similarly, a recent randomized controlled trial by Heinz et al. (2025) using a generative AI therapy chatbot showed significant reductions in symptoms of depression, anxiety, and early psychosis, with participants rating the therapeutic alliance as comparable to that with human therapists.

Concerns regarding AI in mental healthcare are reminiscent of earlier skepticism about internet-based therapies and bibliotherapy; these approaches, initially questioned due to the absence of face-to-face interaction, have since demonstrated their effectiveness in various contexts (Fulmer et al., 2018). This historical perspective, combined with findings from the present study that perceived authorship significantly influences user evaluations, points toward specific directions for future research. One avenue involves exploring AI as a ‘co-pilot’ or supportive tool for therapists, a model pertinent to the widespread use of asynchronous therapist-patient communication in internet-based psychological treatments. In such applications, therapists would retain primary responsibility for patient communication, with AI providing assistance. Further investigation is also warranted to examine outcomes and engagement related to AI-assisted psychological support within other blended care frameworks or low-intensity interventions.

### 4.5 Strengths and limitations

This study's strengths are, first, the empirical insights into differences in empathy, scientific quality, and preference between AI-generated and expert advice, providing a multidimensional evaluation of AI's capacity to replicate expert-like emotional intelligence. Second, blinding participants to authorship helped to mitigate bias and allowed for testing of perceived versus actual authorship effects. Third, using clinicians as raters adds depth to the understanding of AI's ability to replicate expert-like support compared to consumer-based evaluations. Additionally, the study was conducted in accordance with ethical standards and open science principles, with materials available on OSF.io to support transparency and replicability.

The study has limitations that may affect its validity and generalizability. The predominance of texts written by a single expert author (67 %) may not fully represent how expert advice is evaluated in terms of empathy and scientific quality, underscoring the need for diverse authorship in future research. The professional credentials of raters were self-reported and not verified, which could have led to unqualified individuals influencing the results. Further, the use of convenience sampling and the limited information about the sample characteristics limits the generalizability, as this sampling method may not provide an accurate representation of the target population.

## 5 Conclusion

The findings suggest that AI-generated psychological advice is comparable to expert asynchronous written psychological advice in scientific quality and cognitive empathy while surpassing expert advice in emotional and motivational empathy. However, preferences were strongly influenced by perceived authorship, with advice believed to be expert-authored consistently rated more favorably, regardless of its actual source. These results indicate that biases related to perceived authorship may present challenges for the acceptance of AI in mental healthcare. Further research is needed to examine the role of these biases across diverse contexts and explore strategies for their mitigation. Additionally, future studies should investigate the potential of AI tools to enhance clinical decision-making and improve treatment processes in mental healthcare.

## Declaration of ethical data handling

This study did not include an intervention and no personal data was collected, which is why no ethical approval was needed. The study adhered to ethical guidelines by ensuring the privacy rights of all participants were respected. Informed consent was obtained from all participants through a dedicated section in the questionnaire.

## Declaration of Generative Al and Al-assisted technologies in the writing process

During the preparation of this work the author(s) used OpenAI's ChatGPT-4 in order to create the specialized GPT that produced the advice for the comparison. ChatGPT was also used to make small adjustments in the article like shortening to fit the word count and compiling statistical analysis code. After using this tool/service, the authors reviewed and edited the content as needed and take full responsibility for the content of the published article.

## Declaration of Funding Sources

This research did not receive any specific grant from funding agencies in the public, commercial, or non-profit sectors.

## Declaration of competing interest

The authors declare that they have no known competing financial interests or personal relationships that could have appeared to influence the work reported in this paper.

## Data Availability

The data supporting the findings of this study, along with the statistical analysis code, have been deposited in the Open Science Framework (OSF) repository and are openly available at (Zapel et al., 2024).
